# Pattern of Immune Reconstitution Post Allogeneic Stem Cell Transplant: Data From a Resource Constraint Country

**DOI:** 10.7759/cureus.67566

**Published:** 2024-08-23

**Authors:** Uzma Rahim, Raheel Iftikhar, Tariq Ghafoor, Hashim Khan, Awais Siddiq, Hira Tariq, Afzal Khan, Adil Meraj

**Affiliations:** 1 Clinical Hematology, Armed Forces Institute of Bone Marrow Transplant Centre, Rawalpindi, PAK; 2 Hematology and Oncology, Armed Forces Institute of Bone Marrow Transplant Centre/National Institute of Blood and Marrow Transplant, Rawalpindi, PAK; 3 Paediatrics, Armed Forces Institute of Bone Marrow Transplant Centre, Combined Military Hospital, Rawalpindi, PAK; 4 Clinical Hematology, Armed Forces Institute of Bone Marrow Transplant Centre, Combined Military Hospital, Rawalpindi, PAK; 5 Hematology, Armed Forces Institute of Bone Marrow Transplant Centre, Combined Military Hospital, Rawalpindi, PAK; 6 Biostatistics and Epidemiology, Armed Forces Institute of Bone Marrow Transplant Centre/National Institute of Blood and Marrow Transplant, Rawalpindi, PAK; 7 Clinal Hematology and Bone Marrow Transplant, Armed Forces Institute of Bone Marrow Transplant Centre/National Institute of Blood and Marrow Transplant, Rawalpindi, PAK; 8 Anesthesia and Critical Care, Armed Forces Institute of Bone Marrow Transplant Centre/National Institute of Blood and Marrow Transplant, Rawalpindi, PAK

**Keywords:** leukemia, pakistan, immune reconstitution, allogenic, hematopoietic stem cell transplant

## Abstract

Objective: To determine the Pattern of immune reconstitution (IR) post allogeneic stem cell transplant at six months.

Study design: Prospective observational study.

Place and duration of the study: This study was conducted at The Armed Forces Bone Marrow Transplant Centre (AFMBTC) Rawalpindi, Pakistan, from May 2022 to December 2022.

Methodology: After approval from the institutional review board, informed/written consents were taken from patients. All patients (both genders, irrespective of age) undergoing allogeneic hematopoietic stem cell transplant were included in the study. Patients undergoing haplo-identical or autologous bone marrow transplants were excluded from the study. Innate immune reconstitution was checked by complete blood count and adaptive immune reconstitution at six months was checked lymphocyte subset analysis and serum immunoglobulin levels. Data was entered in pre-designed proforma and was analyzed using IBM Corp. Released 2017. IBM SPSS Statistics for Windows, Version 25.0. Armonk, NY: IBM Corp.

Results: This study analyzes 43 patients, including 67% (n=29) males. The median age at the time of HSCT was 11.19 years. Cellular and humoral reconstitution at six months after transplant was used to assess immune reconstitution. Innate immune reconstitution was checked by complete blood count for count recovery (Neutrophil engraftment), and adaptive immune reconstitution (cellular/humoral) at six months was checked lymphocyte subset analysis and serum immunoglobulin levels. By using chi-square, significant associations were found as pre-transplant condition regimen with CD4 (PChi= 0.001), GVHD prophylaxis with CD4 (PChi= 0.04), source of stem cells with CD19 (PChi= 0.008), patient-donor gender disparity with CD19 (PChi= 0.05), GVHD treatment low CD19 (PChi= 0.04), patient-donor relationship with IgA(PChi= 0.007), IgG (PChi= 0.001), and IgM (PChi= 0.001), gender of the patient with IgA (PChi= 0.04).

Conclusion: Our data suggested that at post-transplant six months, there was adequate immune reconstitution except for CD4 and CD4:CD8 ratio. Therefore, post-transplant, six months in Allo-HSCT could be considered for immunization.

## Introduction

Allogeneic hematopoietic stem cell transplantation is a curable modality for many benign and malignant hematological disorders [1]. Following conditioning chemotherapy, donor hematopoietic and immune cells establish new innate and adaptive immune systems that protect the host from infection and relapse [2]. However, immunity is impaired in the initial months and may take years to completely recover [3].

Successful donor-derived immune reconstitution is affected by various factors, including thymic involution of the host, donor age, conditioning regimen, graft type, stem cell dose, donor-recipient disparity, graft-versus-host disease (GVHD) prophylaxis, presence of GVHD, infection, use of steroids, and pre-transplant irradiation [3,4].

Post-allo-HSCT immune reconstitution occurs over approximately two years with different dynamics in which innate immunity (neutrophils, monocytes, and natural killer cells) typically precedes acquired immunity cellular (T- and B-lymphocytes) and humoral immunity [2,5].

Allogeneic HSCT in Pakistan has been carried out for the last three decades [6]. The majority of transplants are carried out for benign disorders, namely aplastic anemia and beta-thalassemia major. Data regarding the pattern of immune reconstitution following stem cell transplants from Pakistan is lacking. This study was conducted to determine the patterns of immune reconstitution after allogeneic stem cell transplants at the largest transplant center in Pakistan.

## Materials and methods

After approval from the institutional review board (IRB-012/AFBMTC/Approval/2022), informed or written consents were obtained from patients or guardians of all study participants for the confidentiality of this study data. This prospective observational study was conducted at the Armed Forces Bone Marrow Transplant Centre/National Institute of Bone Marrow Transplant (AFMBTC/NIBMT) in Rawalpindi, Pakistan, from May 2022 to December 2022.

All patients (both genders, irrespective of age) undergoing matched-related donor allogeneic hematopoietic stem cell transplants were included in the study. Patients undergoing haplo-identical or autologous bone marrow transplants were excluded from the study. Innate immune reconstitution was checked by a complete blood count for count recovery (neutrophil engraftment), and adaptive immune reconstitution (cellular/humoral) at six months was checked by lymphocyte subset analysis and serum immunoglobulin levels.

Data was entered in pre-designed proforma and analyzed using IBM Corp. Released 2017. IBM SPSS Statistics for Windows, Version 25.0. Armonk, NY: IBM Corp. Mean±standard deviation and median with interquartile range were calculated for the quantitative variables. Frequency and percentages were calculated for qualitative variables like the gender of the patient and donor, disease diagnosis, the relationship of the donor with the patient, and post-transplant complications. For univariate analysis, we applied a t-test and one sample sign test to check the relationship between reconstitution and post-transplant patients. At univariable analysis, we applied chi-square to check the association of cellular immune reconstitution and humoral immune reconstitution with pre- and post-HSCT factors. Statistical significance was defined as a p-value <0.05 with a 95% confidence interval.

## Results

From May 2022 to December 2022, 43 patients were enrolled in allogenic HSCT: 29 (67%) male and 14 (29%) female patients (male-to-female ratio, 2:1), and the median age was nine years with an IQR of 6-12 years. Out of all, 10 (23%) had malignant conditions, while 33 (77%) had non-malignant illnesses. Among patients with malignant diseases, only three (30%) patients underwent cranio-spinal radiation before HSCT. The majority were matched sibling donors (brother), 20 (46.5%), followed by matched sibling donors (sister), 17 (39.5%), a fully matched mother, 4 (9%), and a fully matched father, 2 (5%). About half of the patient donors were the same gender; 26 (60.5%) and 17 (39.5%) were mismatched. Myeloablative conditioning (MAC) as a conditioning regimen was used in 34 (79.1%), followed by non-myeloablative (NMA) in 8 (18.6%), and reduced intensity conditioning (RIC) in 1 (2.3%). Graft versus host disease (GVHD) prophylaxis was given, in which ciclosporin (CSA) was received by 7 (16%) patients, CSA+MTX (Methotrexate) by 25 (58%), CSA+MTX+MMF (Mycophenolate Mofetil) by 8 (19%) patients, CSA+MMF by 2 (5%), and CSA+MMF+Steroid by only 1 (2%). The mean TNC (total nuclear cells) and CD34 doses infused into the patients were 5.1±1.2 and 5±2.2, respectively. The median time for granulocyte recovery was 13 days, with an IQR of 12-14 days, whereas the mean time for a self-sustained platelet recovery was 18.4±6.2 days. Acute GVHD was documented in 44 (33%) of recipients, and 29 (67%) suffered viral reactivation post-transplant.

Innate immune reconstitution by complete blood count for count recovery (neutrophil engraftment) and adaptive immune reconstitution (cellular/humoral) at six months after transplant were used to assess immune reconstitution. In cellular reconstitution, the mean/median of WBC was 7.1±2.5 × 109/L (min-max: 2.3-12.5× 109/L), lymphocyte count was 22.6±11 × 109/L (min-max: 2.1-50× 109/L), CD3 cell was 68 × 109/L (IQR:54-80) (min-max: 34-90 × 109/L), CD4 cell was 19 × 109/L (IQR:12-24) (min-max: 4-42 × 109/L), CD8 cell was 40 × 109/L (IQR:27-53) (min-max: 14-67 × 109/L), CD19 cell was 14 × 109/L (IQR:5-24) (min-max: 1-52 × 109/L), NK cell was 7 × 109/L (IQR:5-13) (min-max: 1-26 × 109/L) and the ratio of CD4 to CD8 was 0.4× 109/L (IQR:0.3-0.9) (min-max: 0.06-1.6). In humoral reconstitution, the mean/median of IgA was 1.7±0.8 g/l (min-max: 0-4), IgG was 12.3 g/l (IQR: 9.4-15.1) (min-max: 1-21), and Ig M was 1.1 g/l (IQR: 0.7-1.7) (min-max: 0-18).

By using the sign test, the median of CD3, CD19, IgG, and IgM were normal in transplanted patients except for CD4 and the CD4:CD8 ratio. By using a t-test, the mean of WBC, lymphocyte count, CD8, and IgA were normal in transplanted patients (Table 1).

**Table 1:** Relationship of reconstitution in post-transplant patients Note: t-test and one sample sign test (Binominal test) were applied. P<0.05* is considered significant. WBC: White blood cell, CD: Cluster of differentiation, NK: Natural killer cell, IgG: Immunoglobulin G, IgM: Immunoglobulin M, IgA: Immunoglobulin A

Parameter (Normal ranges)	Median/Mean ± St.d	95% CI		p-value
		Lower	Upper	
WBC (4 - 12)	7.1±2.5	2.3	12.5	0.001*
Lymphocyte Count (13.4 - 31.7%)	22.6 ± 11.1	2.1	50	0.001*
CD3 (49 – 84%)	68	34	90	1.00
CD4 (28 - 53 %)	19	4	42	1.00
CD8 (12 – 38%)	39.7 ± 14.02	35.4	44.04	0.001*
CD19 (6 - 23 %)	14	1	52	0.76
NK (3 - 29 %)	7	1	26	1.00
CD4:CD8 (1.2 - 2.6%)	0.4	0.06	1.6	0.76
IgG (5.3 - 16.5)	12.3	1	21	0.76
IgM (0.5 - 2)	1.1	0	18	1.00
IgA (0.8 - 4)	1.698 ± 0.88	1.43	1.97	0.001*

By using chi-square (Tables 2-4), a significant association of the pre-transplant condition regimen with CD4 was found, in which 33 (87%) MAC, 10 (10%) NMA, and 3 (1%) RIC had a low CD4 count (PChi = 0.001). GVHD prophylaxis showed significant association with CD4, with low CD4 counts corresponding to 25 (66%) CSA+MTX, 6 (16%) CSA+MTX+MMF, 1 (3%) CSA+MMF, and 1 (3%) CSA+MMF+steroid (PChi = 0.04). There was a significant association between the source of stem cells and low CD19, 8 (73% BMH), and 3 (27% BMH+PBSC) with low CD19 cells (PChi = 0.008). There was an association between patient-donor gender disparity and low CD19 cell count, in which 7 (64% of patients with gender disparity had a slow recovery of CD19 cell count (PChi = 0.05). GVHD treatment also showed a significant association with a low CD19 count (PChi = 0.04). The patient-donor relationship was found to be associated with IgA (PChi = 0.007), IgG (PChi = 0.001), and IgM (PChi = 0.001) levels, in which 3 (37%) mothers and 3 (37%) sisters had low IgA levels; however, 3 (60%) patients with a mother as a fully matched donor and 2 (40%) patients with a matched sibling donor sister had low IgG levels, and 2 (100%) patients with a mother as a donor had a low IgM level. Meanwhile, normal IgA, IgG, and IgM levels were found higher in brother and sister donors. The gender of the patient also showed a significant association with IgA levels, with 3 (37% of male patients) and 5 (62% of female patients) having low IgA levels (PChi = 0.04). 

**Table 2:** Association of humoral immune reconstitution with pre- and post-HSCT factors RT: Radiotherapy, TNC: Total nuclear cells, GVHD: Graft versus host disease, MAC: Myeloablative conditioning, NMA: Non-myeloablative, RIC: Reduced intensity conditioning, BMH: Bone marrow harvest, PBSC: Peripheral blood stem cells, CSA: Ciclosporin, MTX: Methotraxate, MMF: Mycophenolate mofetil.

Parameters	Sub-groups	WBC			CD3			CD4		
		>4	<4	p-value	low	Normal	p-value	low	Normal	p-value
		n(%)	n(%)		n(%)	n(%)		n(%)	n(%)	
Age	<12 years	29(74)	4(100)	0.24	6(100)	27(73)	0.14	29(76)	4(80)	0.85
	>12 years	10(26)	0(0)		0(0)	10(27)		9(24)	1(20)	
Sex	Male	26(67)	3(75)	0.73	3(50)	26(70)	0.32	25(66)	4(80)	0.52
	Female	13(33)	1(25)		3(50)	11(30)		13(34)	1(20)	
Relationship	Brother	19(49)	1(25)	0.48	3(50)	17(46)	0.75	17(45)	3(60)	0.79
	Sister	14(35)	3(75)		3(50)	14(38)		15(45)	2(40)	
	Father	2(5)	0(0)		0(0)	2(5)		2(5)	0(0)	
	Mother	4(10)	0(0)		0(0)	4(11)		4(10)	0(0)	
Diagnosis	Malignant	10(26)	0(0)	0.24	1(17)	9(24)	0.68	9(24)	1(20)	0.85
	Non-malignant	29(74)	4(100)		5(83)	28(76)		29(76)	4(80)	
RT	No	36(92)	4(100)	0.56	6(100)	34(92)	0.47	35(92)	5(100)	0.51
	Yes	7(8)	0(0)		0(0)	3(8)		3(8)	0(0)	
Conditioning Regimen	MAC	30(77)	4(100)	0.55	6(100)	28(75.7)	0.39	33(87)	1(20)	0.001*
	NMA	8(20)	0(0)		0(0)	8(22)		4(10)	4(80)	
	RIC	1(3)	0(0)		0(0)	1(3)		1(3)	0(0)	
Source of Stem Cells	BMH	36(92)	3(75)	0.32	6(100)	33(89)	0.69	35(92)	4(80)	0.45
	PBSC	1(3)	0(0)		0(0)	1(3)		1(3)	0(0)	
	BMH+PBSC	2(5)	1(25)		0(0)	3(8)		2(5)	1(20)	
CD34	≥3	29(74)	4(100)	0.24	4(67)	29(78)	0.52	29(76)	4(80)	0.85
	≤3	10(26)	0(0)		2(33)	8(22)		9(24)	1(20)	
TNC	≥5	24(61)	2(50)	0.65	2(33)	24(65)	0.14	22(58)	4(80)	0.34
	≤5	15(39)	2(50)		4(67)	13(35)		16(42)	1(20)	
Gender Disparity	Yes	15(39)	2(50)	0.65	2(33)	15(40)	0.73	15(39)	2(40)	0.98
	No	24(61)	2(50)		4(67)	22(59)		23(60)	3(60)	
GVHD Prophylaxis	CSA	7(18)	0(0)	0.85	0(0)	7(19)	0.28	5(13)	2(40)	0.04*
	CSA, MTX	22(56)	3(75)		6(100)	19(51)		25(66)	0(0)	
	CSA, MTX, MMF	7(18)	1(25)		0(0)	8(22)		6(16)	2(40)	
	CSA, MMF	2(5)	0(0)		0(0)	2(5)		1(3)	1(20)	
	CSA, Steroid, MMF	1(2)	0(0)		0(0)	1(3)		1(3)	0(0)	
Viral Reactivation	Yes	26(67)	3(75)	0.73	4(67)	25(68)	0.96	25(66)	4(80)	0.52
	No	13(33)	1(25)		2(33)	12(32)		13(34)	1(20)	
aGVHD	Yes	17(44)	2(50)	0.80	3(50)	16(43)	0.75	17(45)	2(40)	0.84
	No	22(56)	2(50)		3(50)	21(57)		21(55)	3(60)	
GVHD Treatment	1stLine	12(31)	1(25)	0.79	3(50)	10(27)	0.38	12(32)	1(20)	0.83
	1st+2ndLine	5(13)	1(25)		0(0)	6(16)		5(13)	1(20)	
	Nil	22(56)	2(50)		3(50)	21(57)		21(55)	3(60)	
Note: chi- square was applied, p<0.05* is considered significant.										

**Table 3:** Association of cellular immune reconstitution with pre- and post-HSCT factors RT: Radiotherapy, TNC: Total nuclear cells, GVHD: Graft versus host disease, MAC: Myeloablative conditioning, NMA: Non-myeloablative, RIC: Reduced intensity conditioning, BMH: Bone marrow harvest, PBSC: Peripheral blood stem cells, CSA: Ciclosporin, MTX: Methotraxate, MMF: Mycophenolate mofetil.

Parameters	Sub-groups	CD19			NK		
		low	Normal		low	Normal	
		n(%)	n(%)	p-value	n(%)	n(%)	p-value
Age	<12 years	8(73)	25(78)	0.71	3(75)	30(77)	0.93
	>12 years	3(27)	7(22)		1(25)	9(23)	
Sex	Male	7(64)	22(69)	0.75	3(75)	26(67)	0.73
	Female	4(36)	10(31)		1(25)	13(33)	
Relationship	Brother	4(36)	16(50)	0.49	3(75)	17(44)	0.65
	Sister	5(45)	12(37)		1(25)	16(41)	
	Father	0(0)	2(6)		0(0)	2(5)	
	Mother	2(18)	2(6)		0(0)	4(10)	
Diagnosis	Malignant	2(18)	8(25)	0.64	2(50)	8(20)	0.18
	Non-malignant	9(82)	24(75)		2(50)	31(79)	
RT	No	11(100)	29(91)	0.29	3(75)	37(95)	0.13
	Yes	0(0)	3(9)		1(25)	2(5)	
Conditioning Regimen	MAC	8(73)	26(81)	0.22	3(75)	31(79)	0.90
	NMA	2(18)	6(19)		1(25)	7(18)	
	RIC	1(9)	0(0)		0(0)	1(2)	
Source of Stem Cells	BMH	8(73)	31(97)	0.008*	4(100)	35(90)	0.79
	PBSC	0(0)	1(3)		0(0)	1(2)	
	BMH+PBSC	3(27)	0(0)		0(0)	3(8)	
CD34	≥3	9(82)	24(75)	0.64	2(50)	31(79)	0.18
	≤3	2(18)	8(25)		2(50)	8(20)	
TNC	≥5	6(54)	20(62)	0.64	1(25)	25(64)	0.12
	≤5	5(45)	12(37)		3(75)	14(36)	
Gender Disparity	Yes	7(64)	10(31)	0.05*	2(50)	15(38)	0.65
	No	4(36)	22(69)		2(50)	24(61)	
GVHD Prophylaxis	CSA	1(9)	6(19)	0.10	0(0)	7(18)	0.85
	CSA, MTX	5(45)	20(62)		3(75)	22(56)	
	CSA, MTX, MMF	3(27)	5(16)		1(25)	7(18)	
	CSA, MMF	2(18)	0(0)		0(0)	2(5)	
	CSA, Steroid, MMF	0(0)	1(3)		0(0)	1(2)	
Viral Reactivation	Yes	7(64)	22(69)	0.75	3(75)	26(67)	0.73
	No	4(36)	10(31)		1(25)	13(33)	
aGVHD	Yes	7(64)	12(37)	0.13	2(50)	17(44)	0.80
	No	4(36)	20(62)		2(50)	22(56)	
GVHD Treatment	1stLine	3(27)	10(31)	0.04*	2(50)	11(28)	0.54
	1st+2ndLine	4(36)	2(6)		0(0)	6(15)	
	Nil	4(36)	20(62)		2(50)	22(57)	
Note: chi- square was applied, p<0.05* is considered significant.							

**Table 4:** Association of humoral immune reconstitution with pre- and post-HSCT factors RT: Radiotherapy, TNC: Total nuclear cells, GVHD: Graft versus host disease, MAC: Myeloablative conditioning, NMA: Non-myeloablative, RIC: Reduced intensity conditioning, BMH: Bone marrow harvest, PBSC: Peripheral blood stem cells, CSA: Ciclosporin, MTX: Methotraxate, MMF: Mycophenolate mofetil.

		IgA			IgG			IgM		
Parameters	Sub-groups	low	Normal		low	Normal		low	Normal	
		n(%)	n(%)	p-value	n(%)	n(%)	p-value	n(%)	n(%)	p-value
Age	<12 years	6(75)	27(77)	0.89	5(100)	28(74)	0.19	2(100)	31(76)	0.42
	>12 years	2(25)	8(23)		0(0)	10(26)		0(0)	10(24)	
Sex	Male	3(37)	26(74)	0.04*	2(40)	27(71)	0.16	1(50)	28(68)	0.59
	Female	5(62)	9(26)		3(60)	11(29)		1(50)	13(31)	
Relationship	Brother	1(12)	19(54)	0.007*	0(0)	20(53)	0.001*	0(0)	20(49)	0.001*
	Sister	3(37)	14(40)		2(40)	15(39)		0(0)	17(41)	
	Father	1(12)	1(3)		0(0)	2(5)		0(0)	2(3)	
	Mother	3(37)	1(3)		3(60)	1(2)		2(100)	2(3)	
Diagnosis	Malignant	2(25)	8(23)	0.89	1(20)	9(24)	0.85	0(0)	10(24)	0.42
	Non-malignant	6(75)	27(77)		4(80)	29(76)		2(100)	31(76)	
RT	No	8(100)	32(91)	0.39	5(100)	35(92)	0.51	2(100)	38(93)	0.69
	Yes	0(0)	3(9)		0(0)	3(8)		0(0)	3(7)	
Conditioning Regimen	MAC	7(87)	27(77)	0.77	5(100)	29(76)	0.47	2(100)	32(78)	0.75
	NMA	1(12)	7(20)		0(0)	8(21)		0(0)	8(19)	
	RIC	0(0)	1(3)		0(0)	1(3)		0(0)	1(3)	
Source of Stem Cells	BMH	8(100)	31(89)	0.60	5(100)	34(89)	0.74	2(100)	39(90)	0.89
	PBSC	0(0)	1(3)		0(0)	1(3)		0(0)	1(3)	
	BMH+PBSC	0(0)	3(9)		0(0)	3(8)		0(0)	3(7)	
CD34	≥3	7(87)	26(74)	0.42	4(80)	29(76)	0.85	2(100)	31(76)	0.42
	≤3	1(12)	9(26)		1(20)	9(23)		0(0)	10(24)	
TNC	≥5	5(62)	21(60)	0.89	3(60)	23(60)	0.98	1(50)	25(61)	0.75
	≤5	3(37)	14(40)		2(40)	15(39)		1(50)	16(39)	
Gender Disparity	Yes	3(37)	14(40)	0.89	2(40)	15(39)	0.98	1(50)	16(39)	0.75
	No	5(62)	21(60)		3(60)	23(60)		1(50)	25(61)	
GVHD Prophylaxis	CSA	1(12)	6(17)	0.59	0(0)	7(18)	0.77	0(0)	7(17)	0.80
	CSA, MTX	4(50)	21(60)		4(80)	21(55)		1(50)	24(58)	
	CSA, MTX, MMF	3(37)	5(14)		1(20)	7(18)		1(50)	7(17)	
	CSA, MMF	0(0)	2(6)		0(0)	2(5)		0(0)	2(5)	
	CSA, Steroid, MMF	0(0)	1(3)		0(0)	1(3)		0(0)	1(2)	
aGVHD	Yes	3(37)	16(46)	0.67	2(40)	17(45)	0.84	1(50)	18(44)	0.86
	No	5(62)	19(54)		3(60)	21(55)		1(50)	23(56)	
GVHD Treatment	1stLine	3(37)	10(29)	0.44	2(40)	11(29)	0.61	1(50)	12(29)	0.75
	1st+2ndLine	0(0)	6(17)		0(0)	6(16)		0(0)	6(15)	
	Nil	5(62)	19(54)		3(60)	21(55)		1(50)	23(56)	
Note: chi- square was applied, p<0.05* is considered significant.										

## Discussion

This study describes the pattern of immune reconstitution and its possible association with pre- and post-transplant variables. According to our study, there was an adequate immune recovery at six months post-transplant, except for the CD4 and CD4:CD8 ratios. Our research indicated that the immunity (innate and adaptive) was sufficiently recovered by six months post-transplant.

In the present study, the mean age at the time of HSCT was 11.19 ± 9.4 years, with 33% female and 67% male. Recent multicenter regional data from Italy [7] also reported a median age of around 8.5 years with 60% males and 40% females, which were similar to the findings of this study. In this study, 60.5% matched donor-patient gender and 39.5% mismatched were reported. Similarly, R. Massoud et al. [8] from Germany reported 67% matched and 33% mismatched donor-patient genders. According to the current study, 77% of the patients had non-malignant illnesses, and 23% had malignant conditions. However, AR Belinovski V et al. [9] from Brazil reported 60% non-malignant and 40% malignant conditions undergoing HSCT. Myeloablative, non-myeloablative, and reduced intensity were the conditioning regimens used in our study, accounting for 79.1%, 18.6%, and 2.3%, respectively. Similarly, K Mellgren et al. [10] from Sweden reported myeloablative in 86% of cases, non-myeloablative in 12%, and 2% of SCID patients received anti-thymocyte globulin (ATG) alone as conditioning before transplantation, and no patient underwent reduced-intensity conditioning. GVHD prophylaxis in the current study was CSA in 16%, CSA+MTX in 58%, CSA+MTX+MMF in 19%, CSA+MMF in 5%, and CSA+MMF+Steroid in 2%. Spadea M et al. [7] from Italy reported ATG+CSA+MTX in 52%, ATG+Rituximab in 21%, CSA±MTX in 14%, PTCy+MMF in 9%, and ATG+Steroid+MMF in 4%. The mean TNC and CD34 doses infused into the patients in our study were 5.1±1.2 and 5±2.2, respectively. Brazilian researchers AR Belinovski et al. [9] reported mean TNC and CD34 values of 4.4 x 10⁸/Kg and 4.5 x 106/Kg, respectively, which were in line with our study. In the present study, the average duration for the engraftment of neutrophils and platelets was 13.4±2.1 days and 18.4±6.2 days, respectively. Jacob RP et al. [11] also reported that the median time to neutrophil and platelet engraftment was around 11 days and was 16 days. The 67% post-transplant viral reactivation rate in our study was in line with data reported by Düver F et al. [12]. We discovered that 33% of recipients had acute GVHD, and 67% suffered viral reactivation. Our research yielded an acute GVHD rate of 33%, which was consistent with data reported by Malard, F et al. [13].

According to our study, both innate (neutrophil, monocyte, and NK cell recovery) immunity and adaptive immunity {cellular (B and T cells)} and humoral (immunoglobulins) immunity were adequately achieved, except for the CD4 and CD4:CD8 ratio at six months post-transplant. The present study findings were in line with data reported by Düzelme KH et al. [14] and Mitchell R et al. [15]. Low CD4 was found to be significantly correlated with both the condition regimen and GVHD prophylaxis in this study. Myeloablative conditioning regimens and CSA+MTX as GVHD prophylaxis were primarily linked to low CD4. Low CD19 was significantly associated with the source of stem cells, patient-donor gender disparity, and the use of steroids in the treatment of GVHD. Our study findings related to CD19 cells were in line with data reported by Qin et al. [16], reporting B cell recovery by 6 months post-transplant; van der Maas NG et al. [17], showing B cell reconstitution affected by the source of stem cell transplant; and Singh et al. [18], showing B cell reconstitution at six months post-autologous and nine months post-allogeneic stem cell transplant. Low IgA levels were significantly associated with patient gender, in which females had significantly lower IgA levels than males, and the patient-donor relationship, which showed a significantly low level in a fully matched mother and sister as donors. Low IgG was also significantly associated with the patient-donor relationship and showed significantly low levels in fully matched female donors (mother and sister). The same association of low IgG levels with female donor-to-male recipients was also reported by Norlinet et al. [19], but the same study also reported a strong association of low IgG with patients aged ⩽30 years, type of GVHD prophylaxis, acute GVHD, and decreased survival rate [20], which was contrary to our study. Low IgM showed only association with the mother as a donor. We did not find any significant post-transplant complications associated with a low CD4:CD8 ratio. With the exception of patient-donor gender disparity, patient-donor relationship, and patient gender, none of the patient or donor characteristics, such as age, sex, or diagnosis were significantly associated with immune reconstitution, as also reported by Politikos I et al. [21].

The limitations of our study are the single-center design, small sample size, small subgroups, variable conditioning, and pre-transplant therapy regimens; therefore, results from our study cannot be generalized.

To the best of the authors' knowledge, this was the first published data from Pakistan regarding immune reconstitution following HSCT. This study also had certain limitations. As this was a single-center study with a relatively small sample size, the study’s findings cannot be generalized. It was recommended that all HSCT centers in Pakistan analyze their data under the auspices of the recently formed Pakistan Blood & Marrow Transplant (PBMT) group in order to produce a better result regarding the pattern of immune reconstitution, factors affecting it, and its effect on HSCT outcomes.

## Conclusions

In conclusion, our data suggested that at six months post-transplant, there was adequate immune reconstitution. Some of the factors that affected immune recovery were identified. Among different factors, a significant association between the pre-transplant conditioning regimen, GVHD prophylaxis, source of stem cells, GVHD treatment, patient-donor gender disparity, and delay in immune reconstitution was found. Therefore, post-transplant six-month Allo-HSCT could be considered for immunization.
